# Late-Onset Pharmacological or Dietary Interventions Improve Healthspan and Lifespan in Male and Female Mice

**DOI:** 10.1093/geroni/igaa057.412

**Published:** 2020-12-16

**Authors:** Sarah Mitchell, Michael MacArthur, Alice Kane, Margaret Torrence, Huseyin Mehmet, James Vath, Brendan Manning, James Mitchell

**Affiliations:** 1 Harvard T.H. Chan School of Public Health, Boston, Massachusetts, United States; 2 Harvard Medical School Center for Animal Resources School of Public Health, Boston, Maine, United States; 3 Harvard TH Chan School of Public Health, Boston, Massachusetts, United States; 4 Zafgen, Inc., Boston, Massachusetts, United States; 5 Zafgen, Inc, Boston, Massachusetts, United States; 6 ETH Zurich, Zurich, Massachusetts, Switzerland

## Abstract

While late-onset dietary or pharmacological interventions can extend longevity in rodents, whether or not they can be used to reverse or forestall onset of aging-related symptoms (i.e. frailty) remains untested. Here, we employed three interventions to test this hypothesis. Male and female C57BL/6 mice were randomized to one of four groups: control, 15% calorie restriction (15CR), 0.1% Methionine Restriction (MR)or ZGN1062 (1.5mg/kg, drug in feed) starting at 21mo. Healthspan measurements (mouse clinical frailty index (FI), blood collection, and hematology) were performed every three months, and survival was assessed for all mice. At baseline there were no significant differences in frailty index. After 6mo FI increased consistent with reduced healthspan in control males (0.23□0.01 to 0.34□0.01 A.U, p<0.0001) and females at this age (0.19□0.03 to 0.24□0.01, p<0.0001). Male 15CR, MR and ZGN1062 mice had significantly lower FI scores at 27mo age (15CR: 0.32□0.01, p=0.02; MR: 0.31□0.01, p=0.0009; ZGN1062: 0.30□0.01, p<0.0001). Female mice were less frail than males at 27mo, suggesting sexual dimorphism in the timing of frailty onset in mice. ZGN1062 significantly extended lifespan in males (HR=0.56, p=0.007) and females (HR=0.46, p=0.001). There was a sexual dimorphism in the ability of 15CR and MR to extend lifespan, and a trend towards increased lifespan in males (HR=0.69, p=0.057 and HR=0.71, p=0.09) but not in females. Histological analysis for cause-of-death is ongoing. Taken together these data suggest that a pharmacological intervention associated with weight loss, which may be a more practical therapeutic strategy towards mitigation of age-related healthspan decline than dietary restriction-based interventions.

